# Short-term treatment with dabigatran alters protein expression patterns in a late-stage tau-based Alzheimer's disease mouse model

**DOI:** 10.1016/j.bbrep.2020.100862

**Published:** 2020-11-19

**Authors:** Jaclyn Iannucci, Shelby L. Johnson, Mark Majchrzak, Benjamin J. Barlock, Fatemeh Akhlaghi, Navindra P. Seeram, Abhik Sen, Paula Grammas

**Affiliations:** a George & Anne Ryan Institute for Neuroscience, University of Rhode Island, Kingston, RI, 02881, USA; b Department of Biomedical and Pharmaceutical Sciences, College of Pharmacy, University of Rhode Island, Kingston, RI, 02881, USA; c Bioactive Botanical Research Laboratory, Department of Biomedical and Pharmaceutical Sciences, College of Pharmacy, University of Rhode Island, Kingston, RI, 02881, USA; d Clinical Pharmacokinetics Research Laboratory, Department of Biomedical and Pharmaceutical Sciences, College of Pharmacy, University of Rhode Island, Kingston, RI, 02881, USA

**Keywords:** Alzheimer's disease, Oxidative stress, Thrombin, Cell signaling

## Abstract

Proteins that regulate the coagulation cascade, including thrombin, are elevated in the brains of Alzheimer's disease (AD) patients. While studies using amyloid-based AD transgenic mouse models have implicated thrombin as a protein of interest, the role of thrombin in tau-based animal models has not been explored. The current study aims to determine how inhibiting thrombin could alter oxidative stress, inflammation, and AD-related proteins in a tau-based mouse model, the Tg4510. Aged Tg4510 mice were treated with the direct thrombin inhibitor dabigatran or vehicle for 7 days, brains collected, and western blot and data-independent proteomics using mass spectrometry with SWATH-MS acquisition performed to evaluate proteins related to oxidative stress, intracellular signaling, inflammation, and AD pathology. Dabigatran reduced iNOS, NOX4, and phosphorylation of tau (S396, S416). Additionally, dabigatran treatment increased expression of several signaling proteins related to cell survival and synaptic function. Increasing evidence supports a chronic procoagulant state in AD, highlighting a possible pathogenic role for thrombin. Our data demonstrate that inhibiting thrombin produces alterations in the expression of proteins involved in oxidative stress, inflammation, and AD-related pathology, suggesting that thrombin-mediated signaling affects multiple AD-related pathways providing a potential future therapeutic target.

## Highlights

• Thrombin inhibition with dabigatran reduces markers of oxidative stress *in vivo*.
• Dabigatran treatment reduces tau pathology *in vivo*.
• Dabigatran treatment promotes factors related to cell survival, synaptic function.

## 1 Introduction

Cardiovascular disease and cardiovascular risk factors (CVRFs) are strongly associated with an increased risk of developing dementia, particularly Alzheimer's disease (AD) [1]. While the connection between CVRFs and AD is well-documented, mechanisms whereby these risk factors confer elevated AD risk have not been delineated. It is likely that one or more pathological mediators involved in the progression of cardiovascular diseases contributes to AD pathology. The multifunctional protease thrombin is implicated in the development of atherosclerosis and diabetes, and more recently suggested as a novel mediator in AD [2,3].

Thrombin is a pleiotropic enzyme that triggers a large and diverse number of cellular events, largely through activation of protease-activated receptors (PARs) [4]. Recent evidence indicates that coagulation proteins, including thrombin, also mediate oxidative stress and neuroinflammation, invariant features of neurodegenerative diseases [5]. To that end, thrombin increases NADPH-dependent superoxide anion and hydrogen peroxide production, and injures neurons via microglial release of nitric oxide (NO) [6,7]. In a primarily amyloid-based AD mouse model (3xTgAD), we have previously shown that administration of the thrombin inhibitor dabigatran significantly decreases expression of reactive oxygen species (ROS) and inflammatory proteins in these mice [8].

There is evidence that thrombin is relevant for AD pathology. Specifically, thrombin is expressed in AD-derived, but not control brain microvessels [9], and the thrombin inhibitor protease nexin-1 is reduced in the perivascular space, suggesting that vascular-derived thrombin is increased [10]. Levels of both thrombin and the thrombin receptor PAR-1 are elevated in AD [11]. Thrombin accumulation has also been identified in neurofibrillary tangles, and signaling through the thrombin receptor induces tau aggregation and related hippocampal degeneration [12]. Thrombin's role as a pathological mediator in a tau-based AD model has not yet been explored. Currently, several tauopathy animal models are being used to study AD, including Tg4510, which overexpresses human tau with a P301L mutation at 13:1 versus murine tau [13]. These mice exhibit profound tau pathology and neuronal loss in the hippocampus and cortex, as well as cognitive deficits and metabolic changes that progress with age [14]. Tg4510 mice also display blood vessel abnormalities accompanied by alterations in oxidative and inflammatory markers [15].

Data linking thrombin to oxidative and inflammatory stress as well as tau-related pathology suggest that thrombin could be a target for therapeutic intervention in AD. The objective of the current study is to investigate the potential therapeutic benefits of inhibiting thrombin in a tau-based animal model of AD. In this pilot study, we explore the effects of short-term treatment with direct thrombin inhibitor, dabigatran etexilate, in aged Tg4510 mice. We hypothesize that inhibiting thrombin will reduce oxidative stress and inflammation-related indicators corresponding to an overall reduction in AD-related pathology in the brain.

## 2 Materials and methods

### 2.1 Animals and treatment

Female transgenic Tg4510 AD mice (PF/CamKII-tTA Tg 129 x tetO-MAPT(P301L) Tg (TG2510 TG/TG)) overexpressing human mutant tau (P301L) and background matched controls (PF/CamKII-tTA Tg 129 x tetO-MAPT(P301L) Tg-3 (TG2510 WT/WT)) were a kind gift from MindImmune Therapeutics, Inc. originally obtained from Charles River (Wilmington, MA). Mice were maintained on normal chow (ENVIGO 2020X, Huntingdon, UK) with water available ad libitum. At 15 months of age, mice were treated via oral gavage with vehicle (2.5% DMSO, 2.5% koliphor EL, 90% diH2O) or dabigatran etexilate (100 mg/kg in uniform suspension, Cayman Pharm, Ann Arbor, MI) daily for 7 days (n≥4), and then euthanized. All animal procedures were performed in accordance with NIH “Guide for the Care and Use of Laboratory Animals” and University of Rhode Island Institutional Animal Care and Use Committee (IACUC) guidelines.

### 2.2 Western blot

Whole brains minus the cerebellum were homogenized by sonication (Branson SX150 Sonifier ®, Branson Ultrasonics, Danbury, CT) in phosphate buffered saline (PBS) with protease inhibitors. Samples were resolved in 4–20% poly-acrylamide gel and transferred to nitrocellulose membrane. Membranes were blocked using antibody-specific concentration of Bovine Serum Albumin (BSA) or Milk in Tris-buffered saline + Tween (TBS-T). Primary antibodies for this study include NOX4 (Sigma, MA; 1:1000), iNOS (Abcam, MA; 1:250), and β-Actin (Santa Cruz, CA; 1:10,000). Tau and p-tau species were detected using phosphor-tau family antibody sampler kit (Cell Signaling, MA; 1:500). Bound antibody was detected with infrared secondary antibodies (Li-COR Biosciences, Lincoln, NE). Membranes were imaged using LiCor Odyssey (LI-COR Biosciences, Lincoln, NE), and quantification was done in ImageJ. Values for each protein were normalized to β-actin loading control on the same blot.

### 2.3 Pressure cycling technology-based protein digestion

Whole brain homogenates were digested using pressure cycling technology (PCT) for LC-MS/MS SWATH acquisition following the previously published method with slight modifications [16]. 500 μg of protein sample was spiked with 2 ng of BSA and incubated with dithiothreitol (100 mM) at 90 °C for 15 min, with shaking. Iodoacetamide (200 mM) was added and samples were incubated at room temperature in the dark for 30 min. Protein was then precipitated using ice-cold chloroform, methanol, and water (1:2:1) followed by centrifugation at 13,400 xG for 5 min at 10 °C. The protein pellet was rinsed with methanol and resuspended in 3% w/v sodium deoxycholate (DOC) in 50 mM ammonium bicarbonate. Samples were placed in MicroTubes (Pressure BioSciences Inc, South Easton, MA) with trypsin at a 1:20 ratio of trypsin:protein. Digestion was performed at 55 °C for 75 cycles (50 s at 35kpsi, 10 s at ambient pressure) in a Barocycler NEP2320-45k (Pressure BioSciences Inc). A second digestion was performed by adding fresh trypsin at the same ratio for an additional 60 cycles. Digestion was stopped and DOC was precipitated by the addition of formic acid in acetonitrile at a final percentage of 0.5%. Samples were centrifuged and supernatant was collected for analysis.

### 2.4 Data independent proteomics using mass spectrometry with SWATH-MS acquisition

Mass spectrometry was performed as previously described with minor modifications [16,17]. Samples were analyzed on a SCIEX TripleTOF® 5600 mass spectrometer using a DuoSpray™ ion source (SCIEX, Framingham, MA) coupled to an Acquity HClass UHPLC system (Waters Corp., Milford, MA). Separation was achieved on an Acquity UPLC Peptide BEH C18 column (2.1 x 150 mm, 300 Å, 1.7 μm) with an Acquity VanGuard pre-column (2.1 x 5 mm, 300 Å, 1.7 μm). The column temperature was set to 50 °C and the autosampler was set to 10 °C. Mobile phase A consisted of 99.9% acetonitrile and 0.1% formic acid. Mobile phase B consisted of 99.9% water and 0.1% formic acid. A linear gradient was used with a flow rate of 100 μL/min for 90 min, as previously published [17]. A mixture of trypsin-digested β-galactosidase peptides were used between every 8 samples to calibrate masses and monitor the TOF detector.

Positive ionization mode was used for data dependent acquisition. The mass spectrometer parameters are as follows: gas 1, gas 2, curtain gas, temperature and ion spray voltage floating were 55 psi, 60 psi, 25 psi, 450 °C, 5500 V, Respectively. Declustering potential was 10, collision energy 10 and collision energy spread 15. For data acquisition, a maximum of 50 candidate ions were monitored for each survey scan. All ions had a charge state from 2 to 4. A range of *m/z* 300–1250 was used for exclusion criteria and all ions that had an intensity greater than 25 cps were chosen for MS/MS analysis. The temperature was set at 450 °C and the total cycle time was 3.5 s with a mass tolerance of 50 mDa during the first 0.75 s survey scan.

For SWATH analysis, all parameters were the same as above except for the following: Seventy SWATH windows per cycle were collected over *m/z* 400–1100 with each window size being *m/z* 10 and TOF masses were collected from *m/z* 300 to 1500.

### 2.5 Mass spectrometry data analysis

LC-MS/MS SWATH data was used to generate spectral libraries through ProteinPilot (SCIEX, Framingham, MA). FASTA files for proteins of interest were downloaded from UniProt [18] and imported into Skyline (MacCoss Lab, University of Washington). In Skyline, at least 3 transitions were selected per peptide, and at least 3 peptides per protein were chosen. Once data was analyzed, the MPPreport (MacCoss Lab, University of Washington) was generated and exported to Excel. In Excel, transitions were averaged, and the sum of each peptide was calculated to yield the total area under the curve representative of each protein. These were then standardized to internal standard, BSA.

### 2.6 Statistical analysis

Data from each experiment are expressed as mean ± standard error (SEM), unless otherwise indicated. All tests were performed in GraphPad Prism (version 8.0). For Western blot, data were analyzed for significance using one-way analysis of variance (ANOVA) and multiple comparisons carried out using the post-hoc Bonferroni test. For ratio of phosphorylated tau to total tau, student's t-test was used to determine statistical differences. For LC-MS/MS SWATH data, multiple t-tests were used to determine statistical differences. Statistical significance was determined at *p < 0.05.* Significance for all tests was defined as follows: *p* ≤ 0.05 (*), *p* < 0.01 (**), *p* < 0.001 (***).

## 3 Results & discussion

Increasing evidence has shown that thrombin, and thrombin-related proteins are elevated in the brains of AD patients [11]. Furthermore, studies using amyloid-based AD transgenic mice models have implicated thrombin as a protein of interest [8,19]. Although thrombin accumulation is co-localized with tau aggregation [20], thrombin's potential role in tau-based animal models has not been explored. In the current pilot study, we examined how inhibiting thrombin may alter tau-related pathologic processes.

**Fig. 1:**
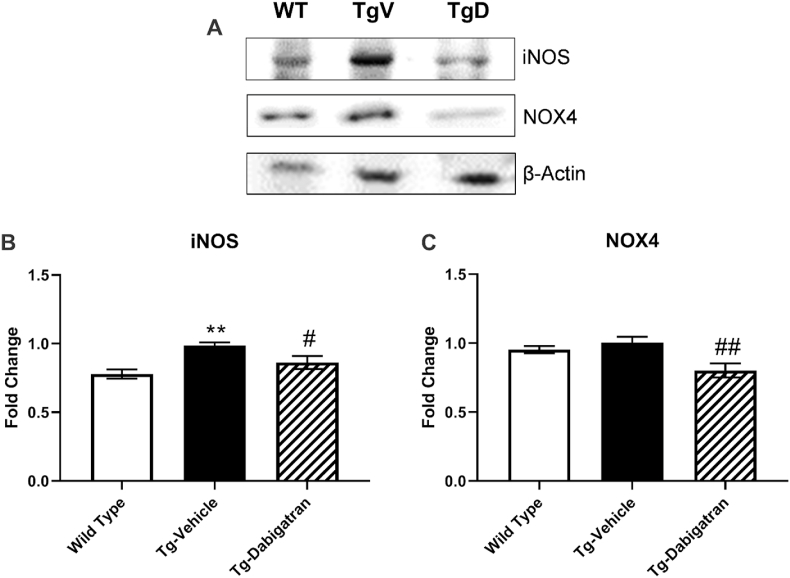
**Dabigatran reduces expression of oxidative stress-related proteins in Tg4510 mouse brains.** Brain homogenates from wild type mice, Tg4510 mice (Tg-Vehicle), and Tg4510 mice treated with dabigatran (Tg-Dabigatran) were analyzed by western blot (A) for iNOS (B) and NOX4 (C). Mean ± SEM (n = 4), significance determined by one-way ANOVA with post-hoc Bonferroni, **p ≤ 0.01 vs. Wild Type, ^#^*p* < 0.05 vs. Tg-Vehicle, ^##^*p* < 0.01 vs. Tg-Vehicle.

Oxidative stress resulting from generation of high levels of ROS in AD is associated with neuron injury, Aβ accumulation, and tau phosphorylation [21,22]. A meditator of several physiologic functions, NO is a pathological mediator in the brain when continuously produced [22], and increased NO and inducible nitric oxide synthase (iNOS) iNOS have been documented in AD models [23,24]. Another source of noxious ROS in the brain is NADPH oxidase (NOX), a family of enzymes that directly regulate ROS production [25]. There is growing evidence that the isoforms NOX1, NOX2, and NOX4 are upregulated in a variety of neurodegenerative disease. Here, we evaluate the expression of both iNOS and NOX4 by western blot (Fig. 1A). The expression of iNOS was significantly different across groups (F = 8.175, *p* < 0.01). iNOS expression was significantly (*p* < 0.01) elevated in Tg4510 mice compared to wild type, and treatment of Tg4510 mice with dabigatran significantly (*p* < 0.05) reduced expression by 17.4% (Fig. 1B). Dabigatran treatment also significantly modified the expression of NOX4 across groups (F = 6.305, *p* < 0.05). Dabigatran treatment significantly (*p* < 0.01) reduced NOX4 expression by 24.7% compared to untreated Tg4510 mice (Fig. 1C). Together, these findings suggest that dabigatran reduces oxidative stress through reduction in enzyme expression.

**Fig. 2:**
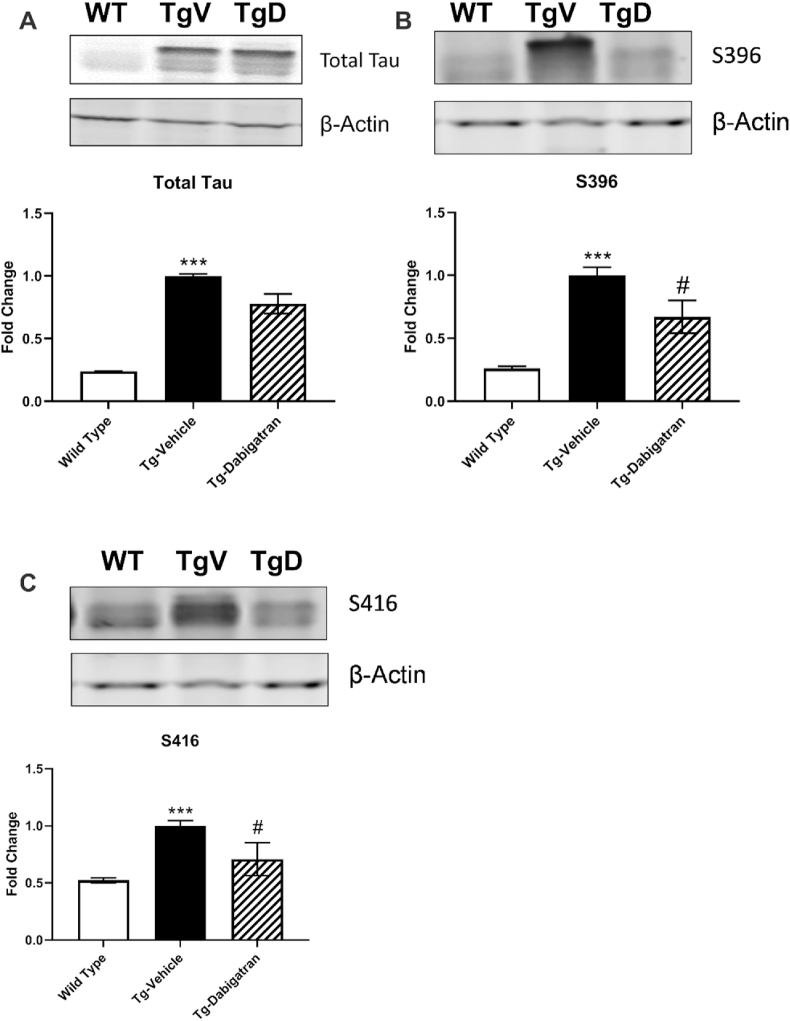
**Dabigatran diminishes levels of phosphorylated tau species in Tg4510 mice.** Brain homogenates from wild type mice, Tg4510 mice (Tg-Vehicle), and Tg4510 mice treated with dabigatran (Tg-Dabigatran) were analyzed by western blot for total tau (A) tau S396 (B) and tau S416 (C). Mean ± SEM (WT n = 5, Tg-Vehicle n = 6, Tg-Dabigatran n = 4), significance determined one-way ANOVA with post-hoc Bonferroni, ***p < 0.001 vs. Wild Type, ^#^*p* ≤ 0.05 vs. Tg-Vehicle.

In order to investigate the effect of dabigatran treatment on tau-related pathology, total tau and phosphorylated forms of tau S396 and S416 were evaluated by western blot (Fig. 2). The expression of total tau (F = 9.942, *p* < 0.01), S396 (F = 26.78, *p* < 0.001), and S416 (F = 11.67, *p* < 0.0015) was significantly different across groups. Tg4510 mice expressed 75.9% more total tau than wild type mice (*p* < 0.001). Expression of both phosphorylated tau species S396 and S416 was significantly (*p* < 0.001) elevated in Tg4510 mice compared to levels in wild type mice. Treatment of Tg4510 mice with dabigatran significantly (*p* < 0.05) lowered levels of both S396 (Fig. 2B) and S416 (Fig. 2C). Furthermore, dabigatran treatment significantly (*p* < 0.05) reduced the ratio of phosphorylated tau to total tau for both S396 and S416 (Table 1). S396 phosphorylation is found early in the disease course of AD [26] and is related to destabilization of microtubules [27]. Phosphorylation at S416 is largely found within the neuronal soma and has been associated with the promotion of AD-related cell death [28]. Together, our findings indicate that dabigatran etexilate treatment may reduce AD-related tau dysfunction through reduced phosphorylation.

**Table 1:** **Ratio of phosphorylated tau to total tau is decreased in Tg4510 mice treated with dabigatran.** Brain homogenates from Tg4510 mice (Tg-Vehicle), and Tg4510 mice treated with dabigatran (Tg-Dabigatran) were analyzed by western blot for total tau, tau S396, and tau S416. The ratio of phosphorylated tau to total tau was calculated for S396 and S416. Values presented as Mean ± SEM. Significance was determined by student's t-test, *p ≤ 0.05.

Tau phosphorylation site	Tg-Vehicle	Tg-Dabigatran	*p* value (significance)
S396	1.010 ± .2061	0.6425 ± .2423	0.0163 (*)
S416	1.005 ± .2938	0.6656 ± .2358	0.0103 (*)

**Fig. 3:**
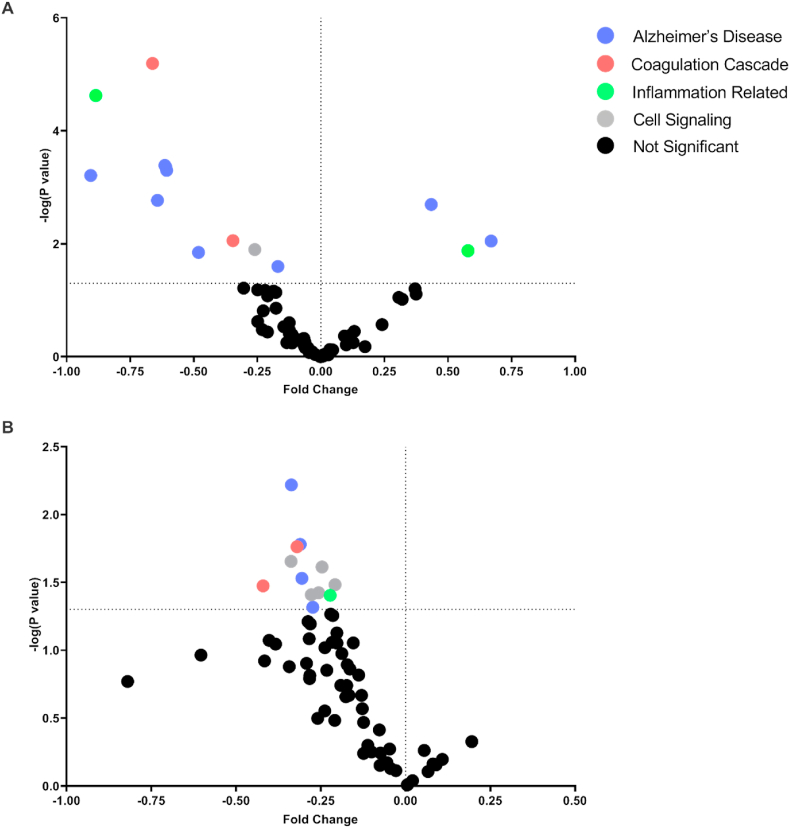
**Proteomic expression differences between wild type mice, Tg-Vehicle, and Tg-Dabigatran mice are detected by LC-MS/MS SWATH analysis.** (A) Volcano plot of data sets from brain homogenates of wild type mice and Tg4510 mice (Tg-Vehicle). (B) Volcano plot of data sets from brain homogenates of Tg4510 mice (Tg-Vehicle) and Tg4510 mice treated with dabigatran (Tg-Dabigatran).

**Fig. 4:**
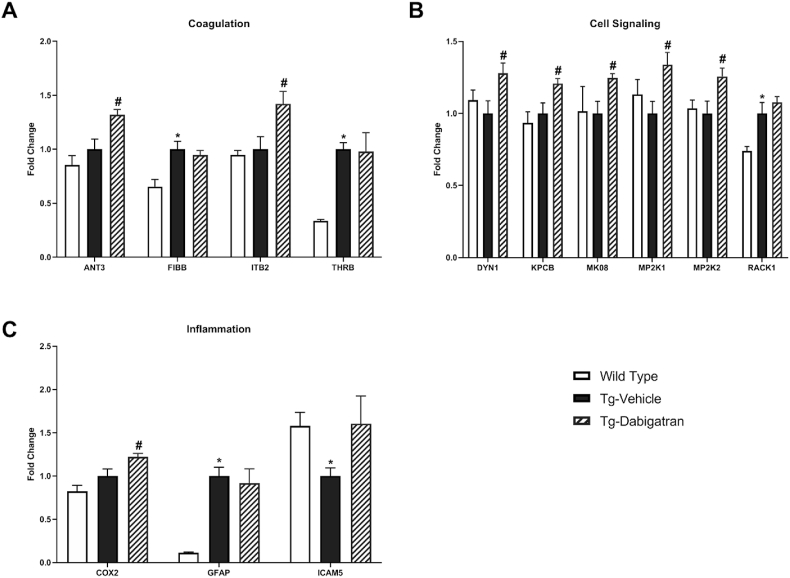
**Treatment of Tg4510 mice with dabigatran causes widespread differences in protein expression.** Proteomic expression differences between wild type mice, Tg4510 mice (Tg-Vehicle), and Tg4510 mice treated with dabigatran (Tg-Dabigatran) are detected by LC-MS/MS SWATH analysis. Functional categories of proteins that show significant expression differences are shown as follows: (A) coagulation cascade, (B) cell signaling, and (C) inflammation-related. Mean ± SEM (n = 5), statistical differences determined by multiple t-tests, *p ≤ 0.05 vs. Wild Type, ^#^*p* ≤ 0.05 vs. Tg-Vehicle.

LC-MS/MS SWATH analysis of all three treatment groups was undertaken based on proteins relevant for AD-related pathological processes as well as thrombin/dabigatran-related mechanisms including proteins involved in the coagulation cascade, cell signaling, inflammation, and AD. Protein sequences were obtained from the UniProt database and results from LC-MS/MS with SWATH acquisition were analyzed in Skyline, an open source software for targeted proteomics (Table 2). Significant differences in protein expression between wild type and Tg4510 mice were identified and visualized with a volcano plot (Fig. 3A). Further LC-MS/MS SWATH analysis was conducted to determine differences in protein expression as a result of dabigatran treatment in the Tg4510 mice, visualized with a volcano plot (Fig. 3B). Significant differences were detected in coagulation proteins (ANT3, FIBB, ITB2, and THRB) (Fig. 4A), cell signaling-related proteins (DYN1, KPCB, MK08, MP2K1, MP2K2, and RACK1) (Fig. 4B), and proteins related to inflammation (COX2, GFAP, and ICAM5) (Fig. 4C).

**Table 2:** **List of proteins and corresponding abbreviations**. Proteins of interest were identified, classified, and categorized based on previous literature. Proteins were obtained from UniProt and data obtained by LC-MS/MS with SWATH acquisition were analyzed in Skyline.

Pathway	Abbreviation	Full Protein Name
Alzheimer's Disease	A4	Amyloid-beta A4 protein (APP)
	ACTN2	Alpha-actinin-2
	ANS1B	Ankyrin repeat and sterile alpha motif domain-containing protein 1B (Amyloid-beta protein intracellular domain-associated protein 1)
	APOE	Apolipoprotein E (Apo-E)
	BACE1	Beta-secretase 1
	BACE2	Beta-secretase 2
	G3BP2	Ras GTPase-activating protein-binding protein 2
	G6PD1	Glucose-6-phosphate 1-dehydrogenase X
	GNAZ	Guanine nucleotide-binding protein G(z) subunit alpha
	HPCA	Neuron-specific calcium-binding protein hippocalcin
	KCC2A	Calcium/calmodulin-dependent protein kinase type II subunit alpha
	NAC2	Sodium/calcium exchanger 2
	NCKP1	Nck-associated protein 1
	PACN1	Protein kinase C and casein kinase substrate in neurons protein 1 (Syndapin-1)
	Q8CFX3	Protocadherin 1
	SV2B	Synaptic vesicle glycoprotein 2B
	SYGP1	Ras/Rap GTPase-activating protein SynGAP
	TAU (Human)	Microtubule-associated protein tau
	TAU (Mouse)	Microtubule-associated protein tau
	TAU (Total)	Microtubule-associated protein tau
	TBB3	Tubulin beta-3 chain
	TLN1	Talin-1
	TTBK2	Tau-tubulin kinase 2
	VIME	Vimentin
Cellular Signaling	CDK5	Cyclin-dependent-like kinase 5
	DYN1	Dynamin-1
	MK01	Mitogen-activated protein kinase 1
	MK03	Mitogen-activated protein kinase 3
	MK08	Mitogen-activated protein kinase 8
	MK09	Mitogen-activated protein kinase 9
	MP2K1	Dual specificity mitogen-activated protein kinase kinase 1
	MP2K2	Dual specificity mitogen-activated protein kinase kinase 2
	KPCA	Protein kinase C alpha type
	KPCB	Protein kinase C beta type
	KPCD	Protein kinase C delta type
	RACK1	Receptor of activated protein C kinase 1
	ROCK1	Rho-associated protein kinase 1
	ROCK2	Rho-associated protein kinase 2
Coagulation Cascade	ANT3	Antithrombin-III
	FA5	Coagulation factor V (Activated protein C cofactor)
	FA8	Coagulation factor VIII (Procoagulant component)
	FA10	Coagulation factor X
	FIBA	Fibrinogen alpha chain
	FIBB	Fibrinogen beta chain
	ITAL	Integrin alpha-L
	ITAM	Integrin alpha-M
	ITAV	Integrin alpha-V
	ITB2	Integrin beta-2
	ITB3	Integrin beta-3
	THRB	Thrombin
Inflammation Related	COX2	Cytochrome c oxidase subunit 2
	DLG4	Disks large homolog 4
	GFAP	Glial fibrillary acidic protein (GFAP)
	ICAM1	Intercellular adhesion molecule 1
	ICAM5	Intercellular adhesion molecule 5
	MILK2	MICAL-like protein 2
	NFKB1	Nuclear factor NF-kappa-B p105 subunit
	NOS3	Nitric oxide synthase, endothelial
	PARK7	Protein/nucleic acid deglycase DJ-1
	PTPRC	Receptor-type tyrosine-protein phosphatase C
	TLR4	Toll-like receptor 4
	VCAM1	Vascular cell adhesion protein 1

Dabigatran treatment significantly increased the levels of a number of cell signaling-related proteins (Fig. 4B). Literature implicates dysfunctional MAPK in neurodegenerative diseases, especially AD. Here, the MAPK-related proteins MP2K1 and MP2K2 were reduced in Tg4510 compared to wild type, and significantly elevated in the brains of dabigatran treated Tg4510 mice compared to vehicle. MP2K1 and MP2K2 are responsible for the activation of the ERK signaling pathway, thought to regulate mechanisms of cell growth, differentiation, and survival [29]. Dabigatran treatment also increased expression of MK08 (JNK1), which is involved in both neuronal degeneration and regeneration, as well as autophagy [30]. Additionally, dabigatran treatment increased expression of dynamin-1 (DYN1), a protein shown to be essential for synaptic vesicle recycling and, hence, for memory formation and information processing [31]. The relationship of both MK08 and DYN1 to autophagy and organelle recycling suggests these pathways should be further explored in light of the literature showing that autophagosome-lysosomal degradation is impaired and AD [32]. Finally, dabigatran treatment increased the level of PKC-β (KPCB), which has been implicated as a critical enzyme in learning and memory function [33]. Elevation of PKC-β is consistent with the increase in DYN1, suggesting that future, long-term studies probing the cognitive effects of dabigatran in this AD model are warranted.

**Fig. 5:**
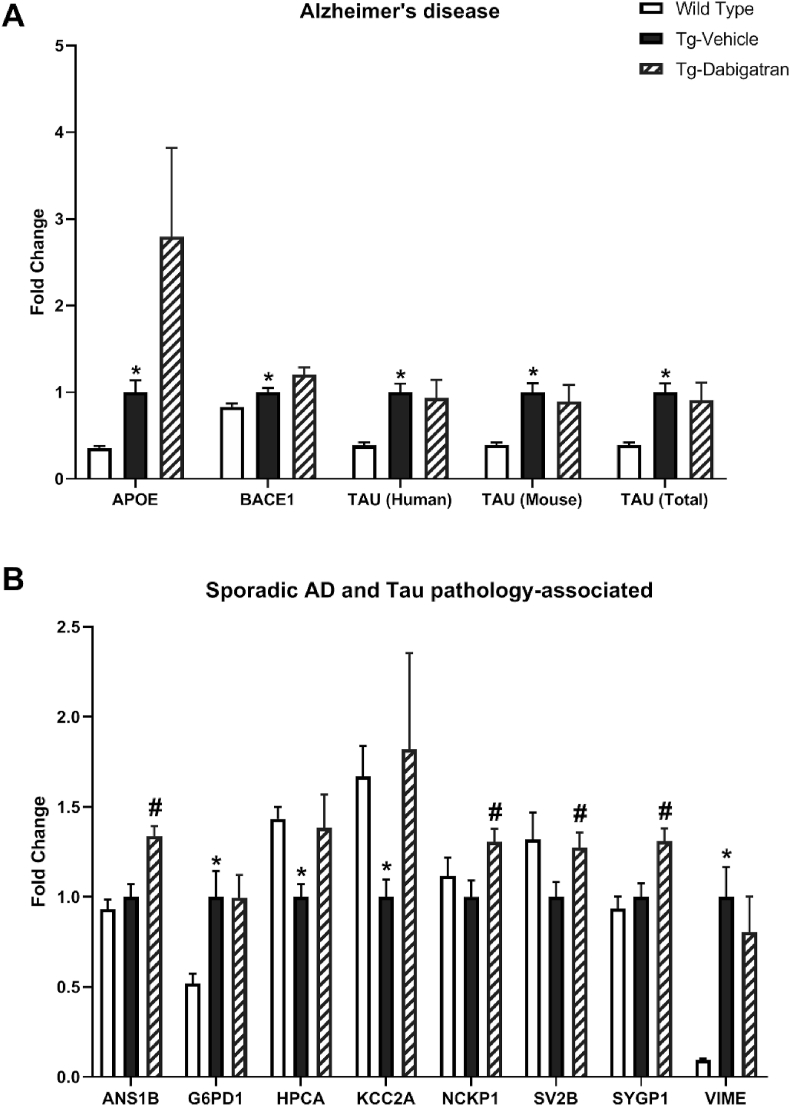
**Treatment of Tg4510 mice with dabigatran alters expression of proteins related to Alzheimer's disease pathology.** Proteomic expression differences between wild type mice, Tg4510 mice (Tg-Vehicle), and Tg4510 mice treated with dabigatran (Tg-Dabigatran) are detected by LC-MS/MS SWATH analysis. (A) classic AD-associated proteins and (B) proteins determined by literature search to be altered in sporadic AD or tau pathology that show significant expression differences are shown. Mean ± SEM (n = 5), statistical differences determined by multiple t-tests, *p ≤ 0.05 vs. Wild Type, ^#^*p* ≤ 0.05 vs. Tg-Vehicle.

To examine AD-related pathology, we performed LC-MS/MS SWATH analysis on a variety of AD-related proteins. A number of classically studied AD-related proteins demonstrated significant differences between wild type and Tg4510 mice, including ApoE, BACE1, TAU (Human), TAU (Mouse), and TAU (total) (Fig. 5A). To further explore AD-related pathology, we performed LC-MS/MS SWATH analysis on a variety of proteins previously found to be altered in sporadic AD and tau pathology, identified through literature review [34]. Here, G6PD1, HPCA, KCC2A, and VIME were found to be significantly different in Tg4510 mice as compared to wild type (Fig. 5B). Dabigatran treatment increased the levels of HPCA and KCC2A to roughly those found in wild type mice. Furthermore, ANS1B, NCKP1, SV2B, and SYGP were significantly increased with dabigatran treatment (Fig. 5B). In a previous report analyzing tau pathology in human AD brains, these proteins were found to be significantly downregulated in late-stage AD [34]. ANS1B (Ankyrin repeat and sterile alpha motif domain-containing protein 1B) is a scaffold protein localized to the post-synaptic density with an identified role in facilitating long-term potentiation (LTP). Additionally, ANS1B has been found to regulate endothelial barrier function and permeability [35]. SV2B (synaptic vesicle glycoprotein 2B) is a mediator of synaptic vesicle transport and exocytosis, and an increase in SV2B expression has previously been tied to behavioral improvements in mice following environmental enrichment [36]. SYGP1 (Ras/RTP GTPase activating protein) regulates the trafficking of α-amino-3-hydroxy-5-methyl-4-isoxazolepropionic acid (AMPA) receptors to the cell membrane, and therefore plays a critical role in regulating neuronal plasticity, synaptic function, and cognition [37,38]. Together, these findings suggest that dabigatran treatment may increase neuronal function at the synapse and promote improved plasticity and learning potential.

When viewing these results, it is important to recognize that dabigatran may exert beneficial effects by mechanisms other than direct inhibition of thrombin-receptor-mediated pathways. Thrombin activity drives the formation of fibrin, and fibrin has been identified as a potential pathological mediator in AD [39]. Anti-coagulant proteins, such as activated protein C (APC), act in opposition to thrombin [40] and promote anti-inflammatory processes through differential activation of the same receptor [41,42]. This may, in part, help to explain the activation of protective MAPK signaling processes following dabigatran treatment. This is supported by the finding that dabigatran treatment significantly increased ANT3 (antithrombin III), a natural thrombin inhibitor (Fig. 4A).

These findings represent preliminary data from a short-term pilot study, as such must be interpreted with a degree of caution. While the expression of both iNOS and NOX4 were significantly decreased with dabigatran treatment, further analysis of enzymatic activity may be required to better understand the anti-oxidant effect of dabigatran. Furthermore, in some cases, as in NOX4, dabigatran significantly alters the impact of a marker that is not significantly changed from wild type to Tg4510 mouse. Without dabigatran treatment in the wild type animals, we cannot definitively state whether the positive effects of dabigatran treatment displayed are specific to the Tg4510 model. Future investigation of the therapeutic benefits of dabigatran treatment should include these relevant control groups, in order to better understand the context in which dabigatran exerts its effects.

Studies in both animal models and human populations utilizing anticoagulant therapies support the notion that mediators of the coagulation cascade may promote AD pathology. A recent study showed that treatment of TgCRND8 transgenic AD mice with dabigatran improved spatial memory deficits and reduced neuroinflammation and amyloid plaque formation [19]. In that study, animals were two months-of-age at the start of the study and received drug treatment for one year. In the current study, we explored the possible acute effects of dabigatran on an older population with established disease. Our results suggest that even a short intervention in older animals produces benefits, as demonstrated by the reduction in oxidative stress and AD markers we document. Future studies are needed to further define the beneficial effects of dabigatran over an extended time course and in additional transgenic models of AD. Our pilot study in a tau-based mouse model adds important information to a growing body of data implicating thrombin inhibition as a therapeutic strategy in AD. An open-label study of a hirudin (natural antithrombin anticoagulant) compound in 84 patients with mild-to-moderate AD found that hirudin plus donepezil reduced the rate of cognitive decline compared to donepezil alone, suggesting that direct thrombin inhibition may indeed be an effective strategy for treating this neurodegenerative disease [43]. In a longitudinal, community-based study, use of dabigatran was associated with a lower risk of new-onset dementia compared to warfarin [44]. Along with our data, these studies demonstrate that targeting thrombin could be beneficial in AD and that thrombin inhibitors could play a role in a drug treatment regimen for AD.

## Funding

This research was funded by 10.13039/100000002National Institutes of Health grant number 1R21NS110628-01; 10.13039/100007625Cure Alzheimer's Fund; and use of the LiCor Odyssey was available through the Rhode Island Institutional Development Award (IDeA) Network of Research Excellence from the National Institute of General Medical Sciences (P20GM103430).

## CRediT author statement

**Jaclyn Iannucci:** Conceptualization, Investigation, Writing (Original & Review/Editing).

**Shelby L. Johnson:** Conceptualization, Investigation, Writing (Original & Review/Editing).

**Mark Majchrzak:** Investigation.

**Benjamin J. Barlock:** Methodology.

**Fatemeh Akhlaghi:** Resources, Supervision.

**Navindra P. Seeram:** Supervision.

**Abhik Sen:** Conceptualization, Supervision.

**Paula Grammas:** Resources, Writing – Review & Editing, Supervision.

## Declaration of competing interest

The authors declare that they have no known competing financial interests or personal relationships that could have appeared to influence the work reported in this paper.
